# SOCS1 and Regulation of Regulatory T Cells Plasticity

**DOI:** 10.1155/2014/943149

**Published:** 2014-07-15

**Authors:** Reiko Takahashi, Akihiko Yoshimura

**Affiliations:** ^1^Division of Rheumatology, Department of Internal Medicine, National Defense Medical College, 3-2 Namiki, Tokorozawa, Saitama 359-8513, Japan; ^2^Department of Microbiology and Immunology, Keio University School of Medicine, 35 Shinanomachi, Shinjuku-ku, Tokyo 160-8582, Japan; ^3^Japan Science and Technology Agency (JST), CREST, Chiyoda-ku, Tokyo 102-0075, Japan

## Abstract

Several reports have suggested that natural regulatory T cells (Tregs) lose Forkhead box P3 (Foxp3) expression and suppression activity under certain inflammatory conditions. Treg plasticity has been studied because it may be associated with the pathogenesis of autoimmunity. Some studies showed that a minor uncommitted Foxp3^+^ T cell population, which lacks hypomethylation at Treg-specific demethylation regions (TSDRs), may convert to effector/helper T cells. Suppressor of cytokine signaling 1 (SOCS1), a negative regulator of cytokine signaling, has been reported to play an important role in Treg cell integrity and function by protecting the cells from excessive inflammatory cytokines. In this review, we discuss Treg plasticity and maintenance of suppression functions in both physiological and pathological settings. In addition, we discuss molecular mechanisms of maintaining Treg plasticity by SOCS1 and other molecules. Such information will be useful for therapy of autoimmune diseases and reinforcement of antitumor immunity.

## 1. Introduction

Dysregulation of immune tolerance to self causes a variety of autoimmune diseases. In the thymus, tolerance is maintained by the so called “negative selection,” deletion of self-reactive T cells. Peripheral tolerance is maintained by the regulatory cells including regulatory T cells (Tregs) [1–4]. Most Tregs mature in the thymus under the influence of relatively high avidity interactions between T cell receptor (TCR) and autoantigens, which are called thymus-derived naturally occurring Tregs (nTregs or tTreg), while some are induced from naïve T cells in the periphery. Tregs consist of 5–10% of CD4^+^ T cells, which express the transcription factor Forkhead transcription factor (Foxp3) in both humans and mice [1]. Foxp3 plays an essential role in the suppressive functions of Tregs [5], and Foxp3 deficiency causes multiorgan autoimmune diseases such as those observed in the scurfy mouse and in patients with immunodysregulation polyendocrinopathy enteropathy X-linked syndrome (IPEX) [6, 7]. Foxp3^+^ Tregs can also be generated from naïve T cells by TCR stimulation in the presence of TGF*β* and IL-2, which are known as induced Tregs or peripheral Tregs (iTregs or pTregs) [8, 9]. Although iTregs and nTregs have similar suppression activity* in vitro*, Foxp3 expression of iTregs has been shown to be unstable* in vivo* [10]. Recently, it has been shown that the terminally differentiated Tregs are not defined entirely by Foxp3 expression, and the natural Foxp3^+^ T cell population is heterogeneous, consisting of a committed Treg lineage and an uncommitted subpopulation with developmental plasticity [11]. This uncommitted subset of Tregs has been shown to lose Foxp3 expression rapidly upon transfer into a lymphopenic host [11] or under inflammatory conditions [12]. This phenomenon, called “Treg plasticity,” has received much attention, because it may play an important role in the pathogenesis of autoimmunity. For example, Komatsu et al. reported that Th17 cells originating from Foxp3^+^ T cells have a key role in the pathogenesis of autoimmune arthritis [13]. Thus, a better understanding of this mechanism is required in order to develop an efficient Treg transfusion therapy for patients with autoimmunity.

In this paper, we review the following: (1) Foxp3^+^ T cell plasticity, particularly under inflammatory conditions, (2) the effect of suppressors of cytokine signaling 1 (SOCS1) deficiency on Foxp3^+^ T cell plasticity, and (3) the effect of Foxp3^+^ T cell plasticity on the possible pathogenesis of autoimmunity, such as systemic lupus erythematosus (SLE).

## 2. Factors Required for Foxp3 Expression

nTregs develop from progenitor CD4^+^CD8^+^ double-positive (DP) T cells as do other single-positive (SP) T cells. TCRs of nTregs are hypothesized to be autoreactive to self-antigens, although Tregs are not deleted [14]. Thus, nTregs are hypothesized to be self-reactive, although no specific self-peptide ligand(s) of an nTreg cell has been identified [15, 16]. In addition to strong TCR signals, the costimulatory receptor CD28 plays an important role in promoting nTreg development. Mice deficient in CD28 or its ligands CD80 and CD86 have significantly reduced nTreg cell populations [17, 18], while deletion of the coinhibitory receptor cytotoxic T lymphocyte antigen (CTLA)-4 results in a higher frequency of nTreg cells [19]. The NF-kB pathway activated by TCR and CD28 plays positive roles in inducing Foxp3, while phosphoinositide 3-kinase (PI3 K) Akt signaling negatively regulates nTreg development [20, 21].

**Figure 1:**
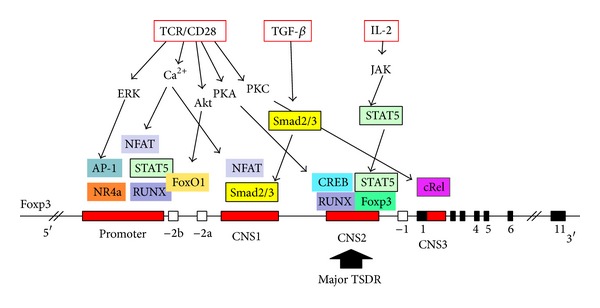
Transcription factors and signals that are involved in Foxp3 induction and stable expression. The promoter and CNSs (conserved noncoding sequences) in the introns are shown. In the course of Treg cell development, epigenetic changes take place and accessibility of CNS2 increases by DNA demethylation, histone modifications, and possibly nucleosome repositioning. The CNS2 region serves as an enhancer for* Foxp3* transcription and is bound by transcription factors such as Foxp3, STAT5, and CREB. These epigenetic alterations are maintained irrespective of environmental changes and thus allow stable* Foxp3* transcription by constitutively expressed transcription factors.

The Foxp3 promoter, which is located 6.5 kb upstream of the first exon, contains six NFAT and AP-1 binding sites as well as a TATA and CAAT box [22]. We recently demonstrated that members of the Nr4a family of nuclear orphan receptors, through their ability to induce Foxp3, are critical in nTreg cell development in the thymus [23, 24]. The promoter is highly conserved between humans, mice, and rats; in addition, three highly conserved noncoding DNA sequences (CNS), CNS1, CNS2, and CNS3, were discovered (Figure 1). CNS1, an intronic enhancer (enhancer 1), contains the TGF-*β*-responsive elements, that is, the Smad2/3 binding sites, close to the NFAT site. These elements are essential for TGF-*β*-induced Foxp3 expression in iTreg cells [25, 26]. Genetic deletion of CNS1 in mice revealed that CNS1 is redundant for nTreg cell differentiation, but essential for iTreg cell generation in gut-associated lymphoid tissues [27]. Consistently, naïve T cells lacking both Smad2 and Smad3 could not differentiate into iTregs [28]. CNS2, corresponding to the TCR-responsive enhancer (enhancer 2), contains a CpG island and binding sites for transcription factors, CREB [29] and STAT5 [30]. Zheng et al. demonstrated that CNS2 is required for Foxp3 expression in mature nTreg cells, while CNS3 acts as a pioneer element, playing a prominent role in the generation of nTreg cells in the thymus and the periphery [27]. CNS3 also contains binding sites for transcription factors such as c-Rel [27]. Major transcription factors found to be involved in Foxp3 gene expression are shown in Figure 1.

## 3. Epigenetic Change in nTregs and Its Role in Treg Stability

Unlike nTregs, TGF-*β* induced Tregs (iTregs) have been shown to be unstable [31–33]. This unstable phenotype is associated with a strong methylation of the CNS2 region of the Treg-specific demethylated regions (TSDRs) within the Foxp3 locus. This idea is supported by the fact that treatment of iTregs with IL-2/anti-IL-2 complexes in the presence of an antigen stabilized Foxp3 expression while also enhancing demethylation of the TSDR [33].

Foxp3 is essential for the development of regulatory T (Treg) cells, yet its expression is insufficient to establish the Treg cell lineage [34]. A recent study has shown that the coexpression of Foxp3 with at least one of the “quintet factors,” namely, the transcription factors GATA-1, IRF4, Lef1, Ikzf4, and Satb1, induces the same pattern of gene expression covering a substantial part of Treg signatures and that this is not achieved by the expression of Foxp3 alone [35]. Ohkura et al. demonstrated that Treg cell development was achieved through a combination of two independent processes, that is, the expression of Foxp3 and the establishment of a Treg cell-specific CpG hypomethylation pattern mostly fond in CNS2 TSDR (nTreg type epigenetics) [36]. This Treg cell-type CpG hypomethylation began in the thymus and spread to the periphery and could be fully established without Foxp3. Hypomethylation of this region was required for Foxp3^+^ T cells to acquire nTreg cell-type gene expression, lineage stability, and full suppressive activity. Thus, those T cells in which the two events have concurrently occurred are developmentally set into the nTreg cell lineage.

Treg epigenetic components control the Treg-type gene expression patterns, either dependent on, or independently from, Foxp3. A genome-wide comparison of DNA methylation status in conventional CD4^+^ T cells and Tregs has demonstrated the presence of Treg-specific DNA hypomethylation in the genes that are associated with Treg function [37]; these genes include Foxp3, Foxp3-dependent Treg cell-associated genes (CTLA4 and GITR), and Foxp3-independent Treg cell-associated genes (Helios and Eos) [38].

## 4. Controversy Surrounding Regulatory T Cell Plasticity

In certain conditions, both murine and human naïve CD4^+^ T cells transiently express Foxp3, without acquiring a suppressive function [39–41]. Moreover, natural Tregs from the thymus have been shown to convert to effector/helper T cells with a decrease of Foxp3 expression [11]. Such “exFoxp3 cells” [12] or “lapsed Tregs” [42] develop an effector-memory phenotype, produce pathogenic cytokines, and might be a cause of autoimmunity. On the contrary, highly purified Tregs are reported to be stable under both physiologic and inflammatory conditions [43]. Regarding the developmental Treg plasticity, two possible mechanisms have been proposed: (1) committed Foxp3^+^ cells convert to Foxp3^−^ cells through lineage reprogramming, and (2) uncommitted Tregs expand and easily lose Foxp3 [44]. A recent study has identified a minor uncommitted nonregulatory Foxp3^+^ T cell population that exhibits transient Foxp3 expression and that lacks TSDR hypomethylation by using fate mapping of mice and methylation analysis [32, 41, 45]. Even committed Tregs are reported to reversibly downregulate Foxp3 expression without losing Treg characteristics [41]. Additionally, CREB and Ets-1 are reported to interact with the CNS2 site of the Foxp3 promoter/enhancer depending on its methylation status and stabilize Foxp3 expression [45–47]. Thus, stable nTregs have a mechanism for stable Foxp3 expression, and a small fraction with unstable Foxp3 expression may contribute to the exFoxp3 population.

In addition to epigenetic modification, protein-protein interactions between Foxp3 and other factors may be involved in Foxp3 stability. Biochemical and mass-spectrometric analyses have revealed that Foxp3 forms complexes with several cofactors [48]. NFAT or Runx1/Cbf*β* binds to Foxp3 in Tregs [49–51]. However, it is not very clear how these Foxp3 binding proteins are involved in Treg functional stability [52].

## 5. Regulatory T Cell Plasticity in Pathological Settings

**Figure 2:**
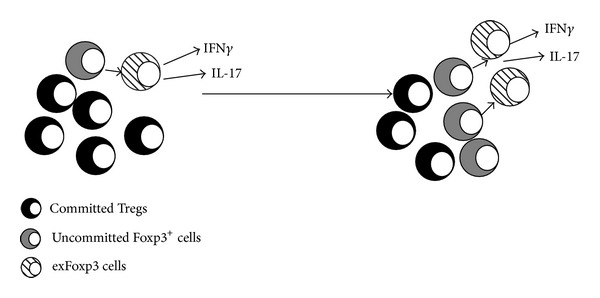
Natural Tregs represent a stable cell lineage; however, there is a minor fraction of Foxp3^+^ T cells that lack Foxp3 expression, which of conversion to exFoxp3 cells is accelerated under lymphopenic or inflammatory conditions.

Conversion of Tregs into pathogenic exFoxp3 cells has been observed under lymphopenic or inflammatory conditions [11, 12, 41, 53–57] (Figure 2). It is important to clarify how exFoxp3 cells that have lost Foxp3 expression can produce proinflammatory cytokines and act as effector cells causing tissue destruction, because there is a high probability that exFoxp3 cells possess autoreactive TCRs [12]. Several reports elucidated that Treg plasticity or IFN*γ*-producing Foxp3 positive cells might be one of the causes of autoimmune diseases or immunological disorders [12, 58–61].

Recently, lineage reporter and tracer mice and Treg cell transfer have shown the association between Treg plasticity and autoimmunity, where Tregs possess antigen-specific TCR within the polyclonal repertoire [62]. A substantial fraction of antigen-specific Tregs with features of Foxp3^high^, CD25^high^, and demethylation of the TSDR, induces downregulation of Foxp3 transcription, loss of Foxp3 expression, and development of effector and pathogenic T cell characteristics in an experimental autoimmune encephalomyelitis (EAE) model. Additionally, the authors emphasized that exFoxp3 cells were apparent, only when this minor fraction was adoptively transferred into mice with ongoing EAE. Another important study showed that IL-17-expressing exFoxp3 cells were differentiated from CD25^low^ Foxp3^+^CD4^+^ T cells then accumulated in the inflamed joints in arthritis [13]. Synovial fibroblasts producing IL-6 caused the conversion of Foxp3^+^CD4^+^ T cells to Th17 cells. These exFoxp3 Th17 cells were elucidated to be more potent osteoclastogenic T cells than conventional Th17 cells derived from naive CD4^+^ T cells, and they expressed Sox4, CCR6, CCL20, IL-23 receptor, and RANKL. Nie et al. also showed that Tregs from RA patients possessed reduced suppression activity due to the dephosphorylation of Foxp3 by TNF-*α*, which is elevated in human RA patients [63]. TNF-*α*-induced Treg cell dysfunction is correlated with increased numbers of Th1 and Th17 cells within the inflamed synovium in rheumatoid arthritis. Another study showed the presence of IFN-*γ* ^+^Foxp3^+^ T cells in MS patients; these double-positive cells acquire a Th1-like phenotype and reduced suppression activity when cultured in the presence of interleukin-12 [58]. These findings establish the pathological importance of a Foxp3^+^ cell subset with unstable Foxp3 in the generation of pathogenic Th cells in human autoimmunity.

## 6. Factors That Control Treg Stability

A Treg transfusion therapy has been successful in animal models of autoimmunity, and Treg therapies are currently being tried in patients [64]. However, Treg cell instability is a concern for developing a Treg therapy because it could cause unexpected adverse effects in patients, and, thus, the factors leading to Treg stability need to be investigated. There must be a mechanism that prevents the pathogenic conversion of nTregs. As mentioned before, hypomethylation of TSDR is a key factor in the stability of Tregs. In addition, several transcription factors and signaling molecules have been shown to be important for Treg stability. Genetic manipulation of genes specifically in Tregs revealed such genes. Not surprisingly, most of these factors are directly or indirectly involved in Foxp3 transcription.

The deletion of Smad2/3 in nTregs resulted in a rapid loss of Foxp3 expression, suggesting that TGF-*β* signals may be necessary for maintaining nTregs in inflamed conditions [28]. The IL-2-STAT5 pathway also seems to be very important for the stability of Foxp3, because the loss of Foxp3 and TSDR methylation can be rescued by means of the enhancement of IL-2 receptor signaling with IL-2-anti-IL-2 complex in EAE [65]. The NF-kB pathway is also important for nTreg stability, probably contributing to Foxp3 transcription through c-Rel, since TRAF6 deficiency in nTregs promoted the loss of Foxp3 expression and Th2 type autoimmunity [66]. Inhibition of p300 (Ep300 or KAT3B), a histone/protein acetyltransferase (HAT) in nTreg cells, destabilized Foxp3 expression and impaired nTreg suppressive function [67]. Foxo1 uniquely regulates nTreg stability, not by sustaining Foxp3 expression but by suppressing genes, including the proinflammatory cytokine IFN-*γ* [68].

Some factors involved in Foxp3 expression have a negative effect. For example, poly (ADP-ribose) polymerase-1 (PARP-1) deficiency in nTregs resulted in stronger suppressive activity and sustained higher expression of Foxp3 and CD25. Thus PARP-1 limits the function of nTregs through modulation of the stable expression of Foxp3 [69]. Deficiencies of C3aR/C5aR signaling augment murine and human iTreg generation, stabilize Foxp3 expression, resist iTreg conversion to IFN-*γ*/TNF-*α*-producing effector T cells, and, as a consequence, limit the clinical expression of graft-versus-host disease [70]. Another factor involved in controlling Treg stability is the ubiquitin-dependent degradation of Foxp3. However, the impact of certain protein modifications to Foxp3, such as ubiquitination and acetylation, on nTreg fate and functions remains to be clarified [71].

## 7. Effects of SOCS1 on Foxp3 Stability and Treg Functions

Inflammatory cytokine signaling including IFN-*γ* and IL-6 signaling plays important roles in the pathogenic conversion of nTregs. Usually,* bona fide* Tregs are expected to be resistant to the effect of such inflammatory cytokines. SOCS1, an inhibitor of cytokine signaling, plays an essential role in maintaining functional nTregs [72–74]. SOCS proteins are the negative regulators of the cytokine-JAK-STAT pathway [75], and uncontrolled IFN*γ* signaling results from SOCS1 deficiency. High expression of SOCS1 in Tregs might be linked to a fundamental function of Tregs. SOCS1 deficiency in Tregs did not affect* in vitro* suppression activity, however, impaired suppressive function of Tregs* in vivo* despite the increase in Tregs. SOCS1-deficient Tregs easily lose Foxp3 expression and converted into Th1- or Th17-like cells, probably due to hyperactivation of STAT1 and STAT3. Recently, Ubc13 has been reported to be involved in suppressive activity by controlling effector cytokine signaling molecules of Tregs including SOCS1 [76].

Analysis of T cell-specific-*Socs1*-conditional knockout (*Lck*Cre-*Socs*1^f/f^,* Lck*Cre-cKO) mice has revealed that SOCS1-deficient effector T cells produce high levels of IFN*γ* and low levels of IL-17 [77]. The defective suppression activity of SOCS1-deficient Tregs from* Lck*Cre-cKO mice was confirmed through the failure to suppress colitis in* Rag2*-deficient mice by the cotransfer of naïve T cells and Tregs. Under lymphopenic conditions, SOCS1-deficient Tregs from* Lck*Cre-cKO mice lost Foxp3 and were converted into Th1 to produce IFN*γ* with accelerated methylation of DNAs in the CNS2 region of the Foxp3 promoter/enhancer. Foxp3 levels were restored in SOCS1^−/−^IFN*γ* ^−/−^Tregs with hypomethylated TSRD.

We propose that STAT1 and STAT3 hyperactivation due to SOCS1-deficiency is the reason for Treg instability and loss of suppressive functions; however, how activated STAT1 and STAT3 affect Foxp3 expression and Treg functions remained to be elucidated. STAT1 may antagonize STAT5, but this is unlikely because we did not observe a reduction in STAT5 phosphorylation in SOCS1-deficient Tregs, instead SOCS1-deficient Tregs expanded well due to stronger IL-2/STAT5 activity [73]. STAT1 has also been shown to inhibit the TGF*β*/Smad pathway [77]. The Smad2/3-deficient Treg phenotypes were similar to those observed in SOCS1-deficient Tregs [28]. Thus, interactive suppression of these molecules by STAT1 may be a mechanism of Foxp3 instability.

Recently, neuropilin-1 (Nrp-1), highly expressed in nTregs but not in iTregs [78, 79], has been implicated in suppressive function of Tregs [28]. Nrp-1 binds semaphorin-4A expressed in mainly plasmacytoid dendritic cells, and this interaction would inhibit Akt-mTOR signaling. High expression of Nrp1 on nTregs might be associated with induction of SOCS1 and Treg plasticity.

**Figure 3:**
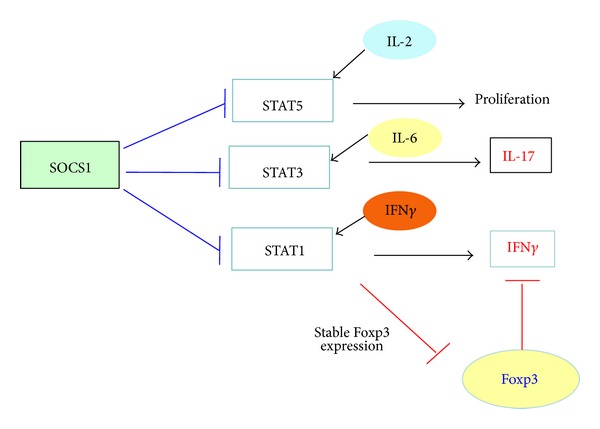
Role of STAT1 and STAT3 in Foxp3 expression and cytokine production in Tregs. SOCS1 protects Tregs from harmful effects of inflammatory cytokines, which promote the loss of Foxp3 expression and the conversion into Th1- and Th17-like effector cells. Hyperactivation of IFN-*γ*-STAT1 pathway results in the loss of Foxp3 expression and hyperproduction of IFN-*γ*. Although STAT3 is not directly involved in Foxp3 expression maintenance, it may be crucial for suppression of the production of IL-17. SOCS1-deficient Tregs may expand faster due to hyperactivation of STAT5.

We propose the possibility that SOCS1 upregulation in Tregs at appropriate levels maintains Treg functions because SOCS1 may protect Tregs from harmful effects of inflammatory cytokines, which accelerates conversion of Tregs into effector cells (Figure 3).

## 8. Regulatory T Cell Plasticity in Systemic Lupus Erythematosus (SLE)

SLE is characterized by dysregulated immunity with both hyperactive T cells and B cells, and terminally pathogenic antibodies construct disease conditions. Dysregulation of Treg functions has been implicated in the pathogenesis of SLE. For example, autoreactive T cell expansion and autoantibody production were accelerated in thymectomized or Treg-depleted lupus-prone mice [80]. Transfer of CD4^+^CD25^+^ Tregs from syngeneic normal mice into SLE model mice can effectively suppress the progress of lupus autoimmune phenotypes, such as increased level of ds-DNA antibody and lupus nephritis [81]. We observed that SOCS1-deficient T cells induce lupus-like autoimmunity including spontaneous dermatitis, splenomegaly, and lymphadenopathy with elevated ds-DNA antibodies [57]. Thus, SOCS1 might be important in the pathogenesis of SLE through Treg plasticity.

It is unclear how Treg dysfunction, including Treg plasticity, causes pathogenic autoantibodies with tissue injuries. Studies have reported that adoptive transfer of Tregs into Cd3*ε* ^−/−^ hosts, which retain B lymphocytes, resulted in the loss of Foxp3 expression and generation of lapsed Tregs that differentiated into follicular helper T cells in Peyer's patches, which promoted IgA class switching [82]. As mentioned, Treg-specific TRAF6-deficient mice possess unstable Tregs and were found to develop SLE-like pathology such as hyperimmunoglobulinemia and anti-dsDNA antibody production [66]. A subset of Foxp3-positive regulatory T cells were recently discovered in the follicular helper T (T_FH_) cell fraction, so called T_FR_ cells. T_FR_ cells are defined as expressing Foxp3, CXCR5, Bcl-6, and PD-1, localizing in the B cell follicles, and controlling the germinal center reactions to produce IgG [83–85]. TRAF3 was shown to be crucial for antigen-stimulated production of T_FR_ cells to mediate ICOS through NF-kB signaling [86]. However, the origin of T_FH_ cells and whether or not it is associated with Treg plasticity still needs to be clarified.

It has been reported that patients with active SLE have a significantly decreased frequency of activated Tregs, and this decrease is correlated with disease activity [87, 88]. Further research in healthy humans and in patients with autoimmune diseases is required to determine associations between Treg plasticity and SLE.

## 9. Conclusions

Evidence on associations between Treg plasticity and pathogenesis of autoimmunity including SLE has been reported. We suggest that an important molecule, SOCS1, prevents acceleration of Treg plasticity and development of autoimmunity. However, mechanisms to control Treg plasticity remain to be clarified. There are also few reports on Treg plasticity in humans. A Treg transfusion treatment for autoimmune patients is now being investigated, and it is necessary to determine and control the Treg plasticity mechanisms.
